# Attentional modulation of speed-change perception in the perifoveal and near-peripheral visual field

**DOI:** 10.1371/journal.pone.0203024

**Published:** 2018-08-30

**Authors:** Taoxi Yang, Hans Strasburger, Ernst Pöppel, Yan Bao

**Affiliations:** 1 School of Psychological and Cognitive Sciences, Peking University, Beijing, China; 2 Institute of Medical Psychology and Human Science Center, Ludwig-Maximilian University, Munich, Germany; 3 Department of Medical Psychology and Medical Sociology, Georg-August University, Göttingen, Germany; 4 Beijing Key Laboratory of Behavior and Mental Health, Peking University, Beijing, China; State University of New York Downstate Medical Center, UNITED STATES

## Abstract

The ability to perceive changes in motion, such as rapid changes of speed, has important ecological significance. We show that exogenous and endogenous attention have different effects on speed-change perception and operate differently in different regions of the visual field. Using a spatial-cueing paradigm, with either exogenous or endogenous cues followed by drifting Gabor patches of changing speed that appear at the cued or uncued location, we measured participants’ thresholds for localizing both acceleration and deceleration of the Gabor patches in different regions (5° and 10°) of the visual field. The results revealed a larger exogenous cueing effect, indexed by a lower threshold for the cued relative to the uncued conditions, at 5° for perceiving acceleration and at 10° for perceiving deceleration. Endogenous attention, in contrast, improved performance equally at both eccentricities. We conclude that exogenous and endogenous spatial orienting constitute two independent attentional systems, with distinct modulation patterns on speed change perception in the visual field. While exogenous attentional modulation is eccentricity-dependent, endogenous attention acts homogeneously in perifoveal and near-peripheral regions of the visual field.

## Introduction

The role of covert spatial attention (i.e., the focusing of attention on a peripheral location without change in gaze) has been well examined in a variety of visual tasks. Spatial attention allows selective prioritization of stimulus processing at a given location, and selectively enhances, amongst others, contrast sensitivity, spatial resolution, reaction time, two-pulse resolution, and processing speed for stimuli at the attended location [1,2,3,4,5]. Besides effects of attention on static stimulus properties, there is evidence that spatial attention also plays a role in the perception of aspects of visual motion, such as motion coherence and the speed or the size of moving stimuli [6,7,8]. These studies have mainly focused on how we perceive constant motion, however, motion change, i.e., acceleration and deceleration, has not yet been studied in detail. Given that objects under ecological conditions rarely move at constant speed, the ability to detect changes in our ever-changing environment is an important challenge for the human visual system, e.g. perceiving transient changes of the speed of a moving object in a specific direction to avoid collision. One goal of the present study, therefore, is to explore the effects of spatial attention on speed-change perception.

A sudden increase or decrease in speed represents an abrupt stimulus change and as such resembles a stimulus onset or offset, where neurons typically respond by an increase or decrease in firing rate [9]. However, unlike stimulus onset or offset, speed changes may require integrating and comparing speed information over time. Thus, processes that mediate responding to a stimulus change may differ from those for the response to stimulus onsets. In addition, there is, (to the best of our knowledge), no unequivocal clear evidence as to whether the brain has a direct representation of the extent of stimulus acceleration or deceleration [10,11,12]. Even though single neurons in area MT apparently encode motion attributes like the motion’s direction and the speed of a moving target, and the neurons’ activity pattern are correlated with behavioral performance during motion detection and discrimination [13,14,15], a similar cellular mechanism for coding acceleration and deceleration apparently does not exist; single neurons in area MT do not seem to be sensitive to acceleration. Alternatively, population pooling of neuronal responses could be a process underlying the representation of changes, and in particular speed changes over time [16,17,18]. Furthermore, considering the aforementioned studies which show that spatial attention enhances several aspects of visual processing, it is worthwhile to determine whether the mechanisms that are possibly responsible for acceleration and deceleration could be modulated by spatial attention.

It has been well-established that covert spatial attention can be uncoupled from gaze [19] often described with the spotlight metaphor [20], and it has been hypothesized since Wilhelm Wundt [21] more than a hundred years ago that there are at least two ways of allocating spatial attention. On the one hand, observers can voluntarily deploy attention to the spatial location that is relevant for a current task. On the other hand, physically salient stimuli can involuntarily capture attention, even when they are unrelated to the current goal-directed task. In the spatial cueing paradigms developed by Eriksen and Posner and colleagues [20,22,23,24] spatial attention can be either attracted exogenously and reflexively, using a spatially non-predictive peripheral cue, or directed endogenously and voluntarily, using a spatially predictive central symbolic cue. Similar distinctions have been conceptualized as automatic vs. voluntary attention [25] or transient vs. sustained attention [26]. Attentional modulation of performance typically differs depending on the type of spatial attention deployed [25,26,27,28,29] (see [1] for review). Thus, the present research asks further whether and how these two types of attention affect speed-change perception.

It has become increasingly evident that attentional effects also show functional differences in different regions of the visual field. That might not come as a surprise in light of the fact that perceptual performance shows essential inhomogeneities in the visual field [30,31] most notably between the central region and the more peripheral visual field (see [32] for review). Using a spatial cueing paradigm with inhibition of return (IOR, the inhibitory process setting-in approximately 300 ms after the initial visual orienting at the cued location; cf. [33,34]), Bao and colleagues found in a series of studies that spatial-cueing effects varied as a function of stimulus eccentricity; more specifically, the inhibitory component of attentional control, indexed by the magnitude of IOR, is much stronger at the periphery than the perifoveal region suggesting different processing mechanisms for perifoveal and peripheral stimuli [35,36,37,38]. This eccentricity effect is further found to be independent of cortical magnification [39] and it is resistant to subjects’ practice [40]. These findings provide support for the hypothesis that attentional modulation in the visual field is not homogeneous. Therefore, it is reasonable to hypothesize that attentional control also operates differently on speed-change perception in different regions of the visual field.

In the experiments presented here, we examined the possible attentional modulation of both exogenous and endogenous attention in a speed-change detection task where targets were presented in different regions of the visual field. It was expected that shifting attention to a specific spatial location would affect the detection of a sudden speed change of moving stimuli at that location. Given the inhomogeneity of attentional control in the visual filed, it was further anticipated that the attentional modulation on speed-change detection would depend on the eccentricity at which the target appears. In Experiment 1, thresholds for localizing both acceleration and deceleration of drifting Gabor patches were measured using a peripheral cueing paradigm in which the Gabor patches that changed speed could appear at two eccentric (5° or 10°), cued or uncued locations. Experiment 2 was designed to investigate whether endogenous attention would affect speed-change perception, and if so, whether its effect would be similar to that of exogenous attention.

## Materials and methods

### Participants

Thirty-six healthy right-handed students from Peking University (aged 18–28 years) participated in the study (Exp 1: 16 participants, 7 males; Exp 2: 20 participants, 10 males). All participants had normal or corrected-to-normal vision, and all were naïve with respect to the purpose of the experiment. All participants gave written informed consent before the experiments and they received moderate rewards for their participation. The experiments had been approved by the Committee for Protecting Human and Animal Subjects in the School of Psychological and Cognitive Sciences at Peking University and were in accordance with the Declaration of Helsinki.

### Apparatus

Visual stimuli were generated using MATLAB 7.13 (MathWorks, Natick, MA) in conjunction with the Cogent toolbox (http://www.vislab.ucl.ac.uk/Cogent/) and displayed on a gamma-corrected 20-in CRT monitor with 1024×768 resolution at a refresh rate of 100 Hz. Responses were collected on a keyboard.

### Materials

In both experiments, stimuli consisted of two diagonally oriented Gabor patches (45° or 135° orientation, spatial frequency:1 cyc/deg, Gaussian envelope in cosine phase with σ = 0.75°, corresponding to a visible grating diameter of ~ 4.5°; Michelson contrast 99.8%; background luminance 20 cd/m^2^), with its grating moving behind the Gaussian aperture orthogonally to their respective orientation, either up-right or up-left. Motion, and motion direction, were clearly visible at all tested conditions. The fixation mark was a white cross subtending 0.8° visual angle. In Exp 1, the spatial cue for attracting exogenous attention was a white dot, presented at 5° or 10° eccentricity to the left or right of the center and subtending 1° visual angle. For Exp 2, a white 0.8°×0.6° arrow, presented in the center of the computer screen, served as endogenous cue.

### Procedure

**Fig 1:**
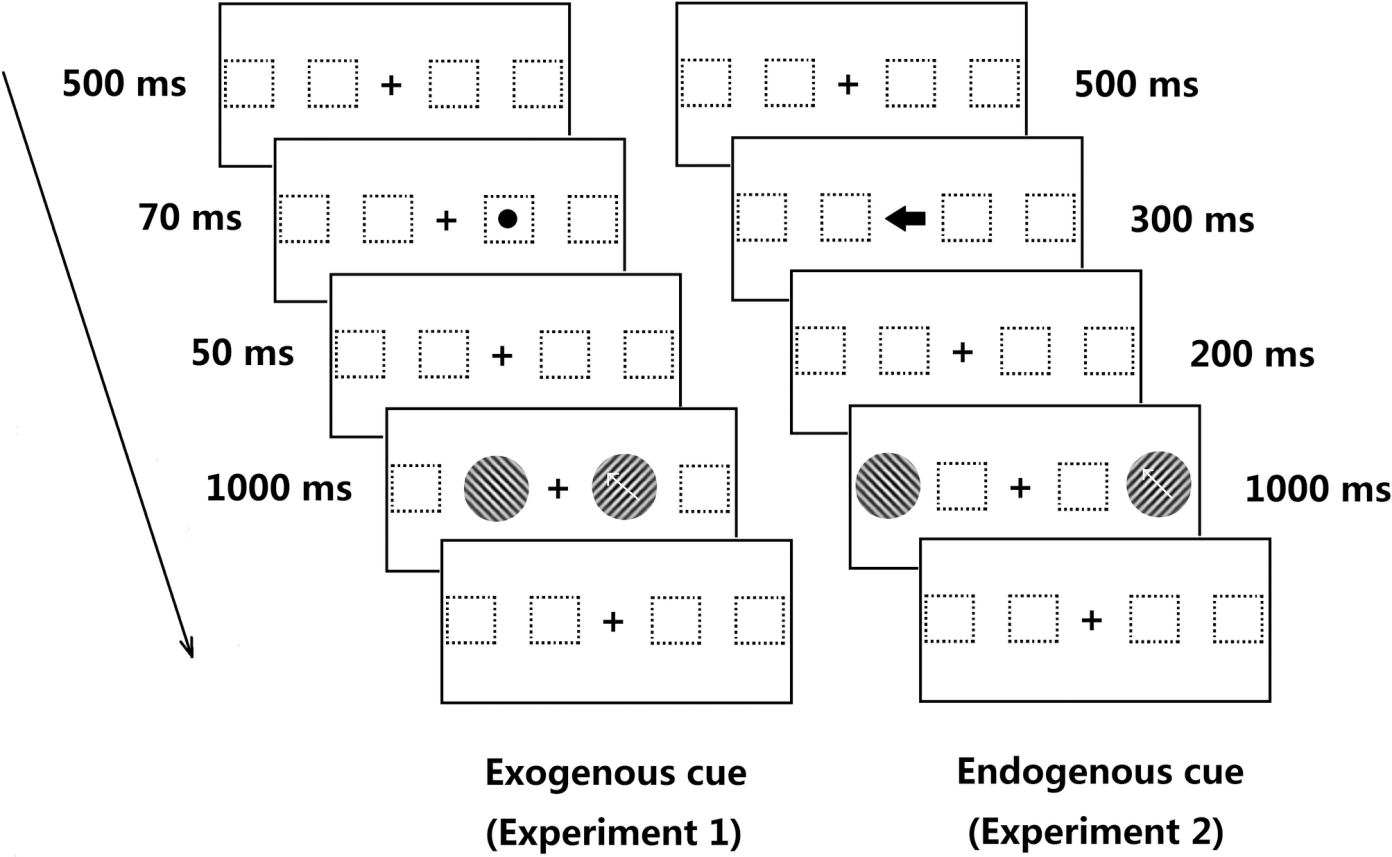
Sample trial sequences in Exp 1 (exogenous cue; left) and Exp 2 (endogenous cue; right). In this example, the target is presented in the cued location at 5° eccentricity (Exp 1) and in the uncued location at 10° (Exp 2).

The experiment took place in a dimly lit room. Subjects were seated at 57 cm distance from a computer screen, with viewing distance kept constant by using an adjustable head-chin rest. The stimulus sequence in a trial is illustrated in Fig 1. Each trial started with the presentation of a fixation point for 500 ms at the center of the screen. In Exp 1, the exogenous cue appeared for 70 ms left or right of the fixation point (50% validity, non-informative) at an eccentricity of either 5° or 10°. An inter-stimulus interval (ISI) of 50 ms followed the cue offset. This interval was chosen short enough to prevent goal-directed saccades. In Exp 2, the endogenous cue was a single arrow pointing either to the left or to the right, indicating the likely location of the changing-speed target (75% validity; that is, the target appeared in the direction of the arrow on 75% of the trials). After the offset of the spatial cue (exogenous or endogenous) and the corresponding ISI, two drifting Gabor patches with a base speed of 6 deg/s were presented, one to the left and one to the right of fixation on the horizontal meridian, at either 5° or 10° of eccentricity. After a fixed-speed period of 500 ms, one Gabor patch abruptly changed its speed, after which the stimulus moved constantly at one of six different test speeds (plus a control) for another 500 ms while the other Gabor patch continued to move at the base speed of 6 deg/s. Speed changes of 0%, ±10%, ±20%, ±30%, ±40%, ±50%, and ±60% relative to the base speed were used; for example, 20% acceleration for 6 deg/s base speed resulted in 7.2 deg/s speed, and 20% deceleration in 5 deg/s speed. Participants were asked to maintain fixation throughout the trial sequence and to indicate, by pressing one of the two keyboard buttons, whether the right or the left stimulus changed its speed. A training phase with neutral-cue trials preceded the experiments.

### Design

The sign of the speed change, i.e., acceleration vs. deceleration, was designed as a between-subject factor; half of the subjects participated in the acceleration condition (8 in Exp 1, and 10 in Exp 2) and the other half in the deceleration condition. In Exp 1, all the other conditions (stimulus eccentricity, left/right visual field, and cue validity) were within-subject factors and were used equiprobably with each of the six speed steps. The resulting 48 different trials (6 speed steps × 2 left/right visual field × 2 eccentricities × 2 cue validities) were repeated 20 times in randomized order, for a total of 960 trials for each subject. In Exp 2, there were 1152 trials for each subject, divided into 24 blocks of 48 trials, of which 36 trials were valid (target appeared at the cued location), and the remaining 12 were invalid. In both experiments, the two eccentricity conditions (5° and 10°) were completed in different sessions and on different days. The condition order was randomized and counterbalanced across participants, i.e. half of the participants performed the 5° condition first, and the other half the 10° condition first.

### Data analysis

Speed-change detection thresholds were obtained by the method of constant stimuli. Psychometric functions were fitted using the Palamedes toolbox for MATLAB [41,42], which implements maximum-likelihood estimation. We fitted the psychometric function with a Weibull function:

$$p_{c}=1‑(1‑\gamma )\;e^{{‑(\frac{x}{\alpha })}^{\beta }},$$

Where *p*_*c*_ is percent correct of detecting the speed change, and *x* is speed-change extent (in percent of the base speed). Parameters α and β determine the threshold and maximum slope of the function, respectively [43]; γ is the performance expected at chance (0.5 in our case of a 2AFC). The *lapse rate* (upper asymptote) was constrained to be unequal to 0 and below 0.1, to avoid a biased estimate of the threshold [44]. The value of α, i.e., the stimulus intensity that is at the curve’s point of inflection and predicts 81.6% correct performance, was defined as the detection threshold [43].

Goodness-of-fit for the psychometric functions was estimated using the function in the Palamedes toolbox, based on Wichmann and Hill [44]. The number of bootstrap simulations performed to determine the goodness-of-fit was 500. To test for influences of attention on performance, model comparisons were performed between the model based on the data of both conditions (valid and invalid cue) and the one treating the two conditions separately, using the Palamedes routine *PAL_PFLR_ModelComparison*. Transformed likelihood ratios of respective pairs of models were taken to be significantly different if exceeding 95% of transformed likelihood ratios obtained through Monte-Carlo simulations (10,000 simulations in each comparison). To quantify and further investigate attentional effects, subjects’ speed-change detection thresholds were submitted to a three-way mixed-design ANOVA (Variables: *sign of speed change*, *stimulus eccentricity*, and *cue validity*). All standard statistical tests and descriptive statistics were performed with SPSS 18.0.

## Results

### Experiment 1

**Fig 2:**
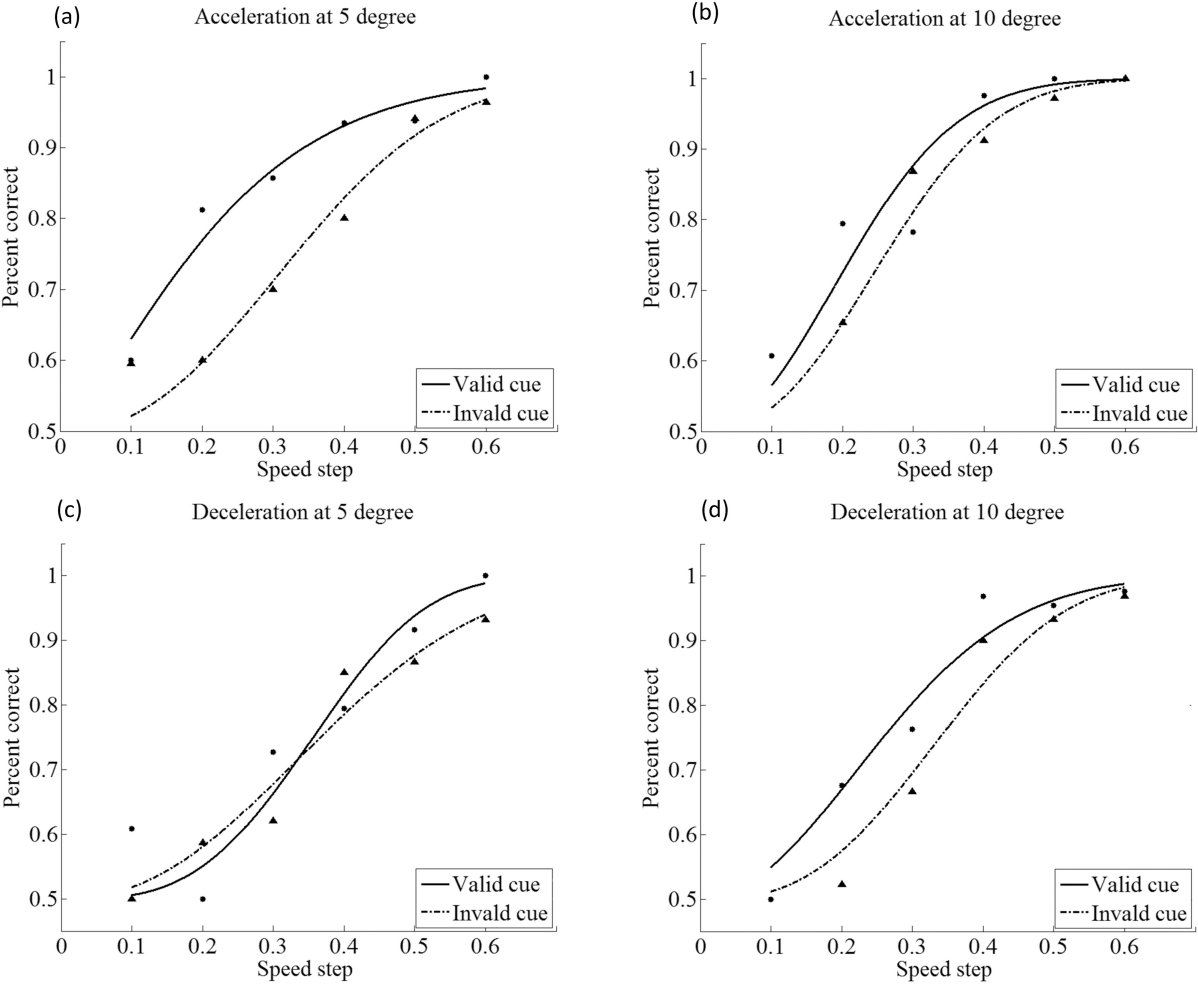
Effects of exogenous attention on performance (percent correctness) as a function of speed change step in Exp1. Solid lines are curve fittings for the valid-cue condition; dashed lines for invalid-cue condition. Acceleration is shown on top panels a and b, and deceleration in bottom panels c and d.

For each speed-change condition (acceleration and deceleration), a psychometric function was fitted to the detection rates of each subject, for 5° and 10° eccentricity, resulting in a total of 72 psychometric function fits (64 at the individual level and 8 at group level). Of these, 68 fits (94.4%) passed a goodness-of-fit test at the 5% level. Fig 2 shows the psychometric functions measured for one subject in our speed change detection task. Trials were collapsed across location (left and right) of the cue and target, and were classified as having a valid or an invalid cue (depending on whether or not the Gabor patches that changed speed indeed appeared at the cued location). When the target was cued, the valid-cue curve shifted to the left of the invalid-cue curve. Under the acceleration condition, the valid-cue and invalid-cue curves were significantly different from each other at both 5° and 10° eccentricity, as assessed by model comparison statistics (both *p* < 0.05). With deceleration, in contrast, the difference between valid-cue and invalid-cue curves did not reach significance at 5° (*p* = 0.34), whereas a significant difference was obtained at 10° eccentricity (*p* < 0.05).

**Fig 3:**
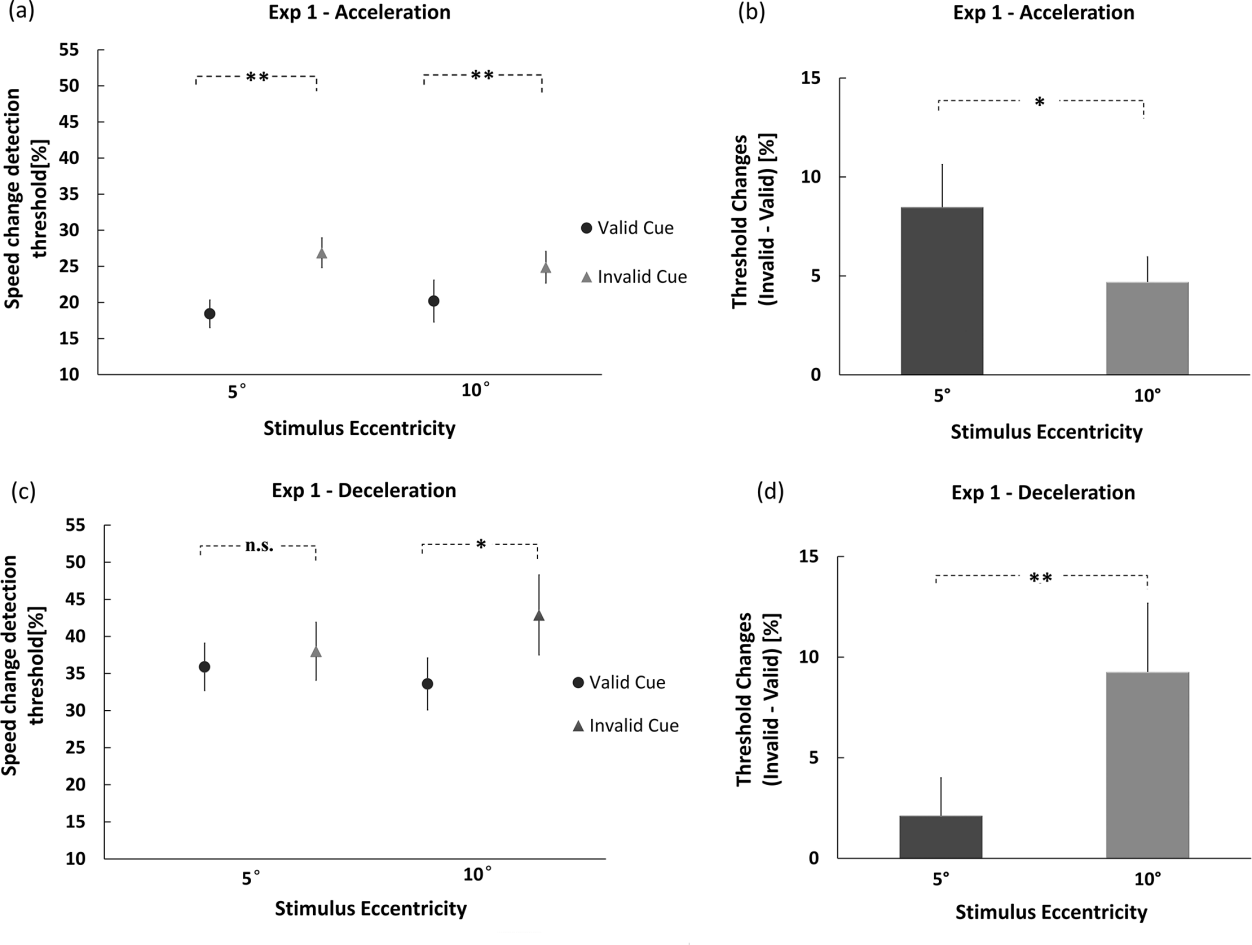
Effects of exogenous attention as indicated by speed-change thresholds in Exp 1. **(**a) The mean speed-change detection thresholds for acceleration in the valid-cue and invalid-cue conditions at 5° and 10°; (b) a larger exogenous cueing effect was observed at 5° under the acceleration condition; (c) the mean speed-change detection thresholds for deceleration in the valid-cue and invalid-cue conditions at 5° and 10°; (d) a larger exogenous cueing effect was obtained at 10° when the speed change was negative (* *p* < .05; ** *p* < .01; Error bars represent the standard error).

Fig 3A and 3C plot the mean speed-change detection thresholds for the valid-cue and invalid-cue conditions at 5° and 10°. The detection thresholds of the participants were analyzed using a 2×2×2 mixed ANOVA, with sign of speed change (acceleration or deceleration) as a between-subject factor, and cue validity (valid or invalid) and eccentricity (5° or 10°) as within-subject factors. Overall, there was a rather large and significant main effect of cue validity, *F*(1,14) = 17.538, *p* < 0.01, $\eta_{p}^{2}$ = 0.556. Detection threshold for the attended target was much lower than for the unattended target, showing a facilitation effect of exogenous attention. The main effect of sign of speed change was also large and significant, *F*(1,14) = 14.691, *p* < 0.01, $\eta_{p}^{2}$ = 0.512. The threshold to detect a deceleration was higher than that of acceleration (37.6% vs. 22.6%). Critically, a significant three-way interaction of sign of speed change, cue validity and eccentricity was observed, *F*(1,14) = 13.710, *p* < 0.01, $\eta_{p}^{2}$ = 0.495. To further explore the interaction, we performed 2×2 ANOVAs for acceleration and deceleration separately, with cue validity and eccentricity as independent variables. When the speed change was positive, the speed-change detection thresholds for the attended and unattended target, respectively, were 18.4% and 26.9% at 5° eccentricity, and 20.2% and 24.9% at 10°. In both pairs were the values significantly different (each *p* value < 0.01). Further paired *t* tests for the detection-threshold difference (unattended–attended) at 5° and 10° eccentricity indicated a larger exogenous cueing effect at 5° eccentricity than at 10° (*p* < 0.05; Fig 3B). In the deceleration condition, the difference between attended (35.9%) and unattended (38.0%) target was only 2.1% at the 5° condition, but was 9.3% at 10° eccentricity (33.6% vs. 42.9%). Only the latter difference was significant (*p* < 0.05). Further comparison of these two speed-detection threshold differences (unattended–attended) at 5° and 10° eccentricity showed that the difference under the 10° condition was significantly larger than that at 5° (*p* < 0.01; Fig 3D).

### Experiment 2

**Fig 4:**
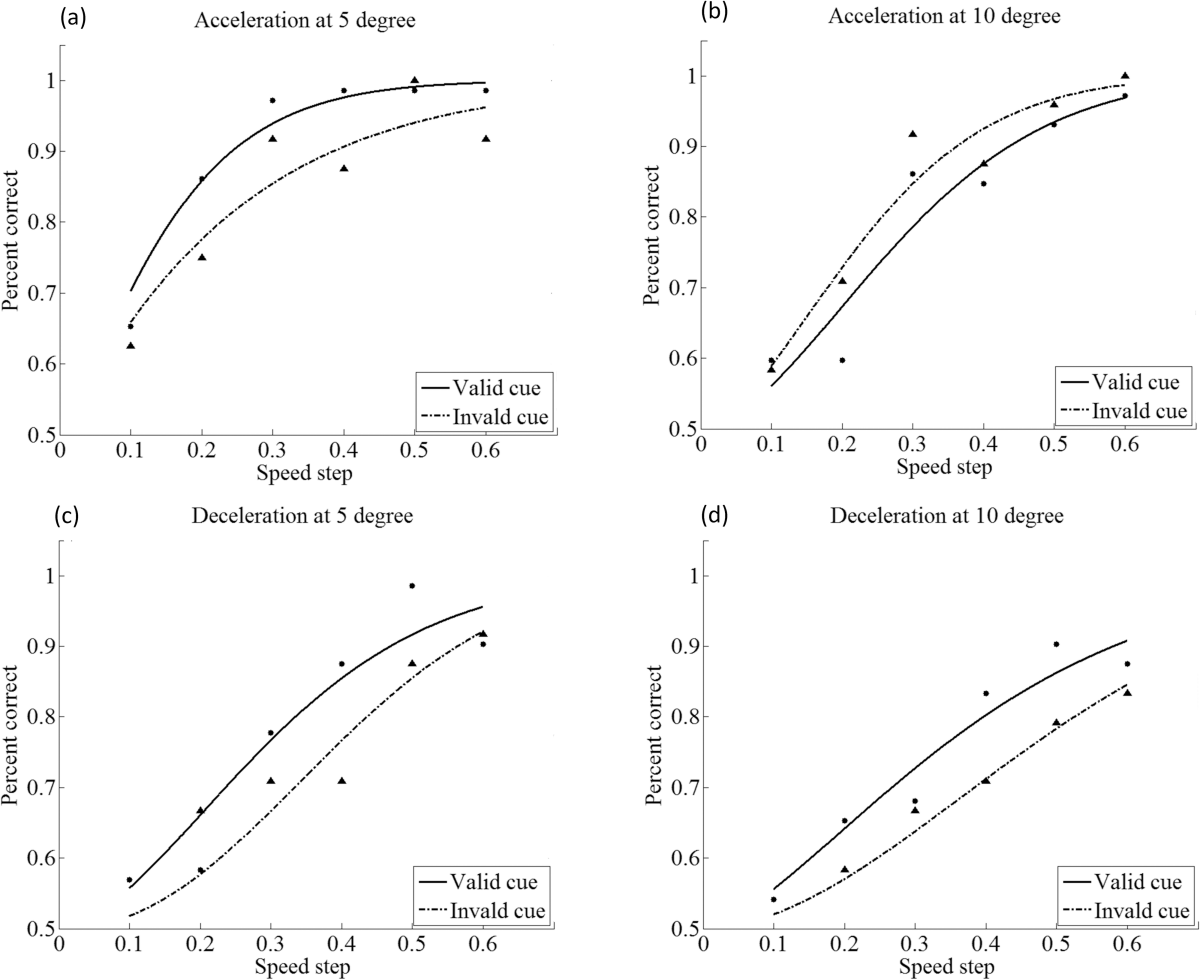
Effects of endogenous attention on the psychometric functions for speed-change detection in Exp 2. Solid lines are curve fittings for the valid-cue condition, dashed lines for the invalid-cue condition. Acceleration is shown on top panels a and b, and deceleration on bottom panels c and d.

Fitting the data to psychometric functions for the various conditions in Exp 2 followed the same procedures as in Exp 1. Among a total of 88 psychometric function fits obtained in Exp 2, there were 80 (i.e., 90.9%) that passed the goodness-of-fit test at the 5% level. Fig 4 shows the psychometric functions measured for one subject in the speed change detection task with endogenous cues. Significantly differing fits of valid-cue curves as opposed to invalid-cue curves by model comparison statistics were observed, both at 5° and 10° eccentricity (all *p* < 0.05).

**Fig 5:**
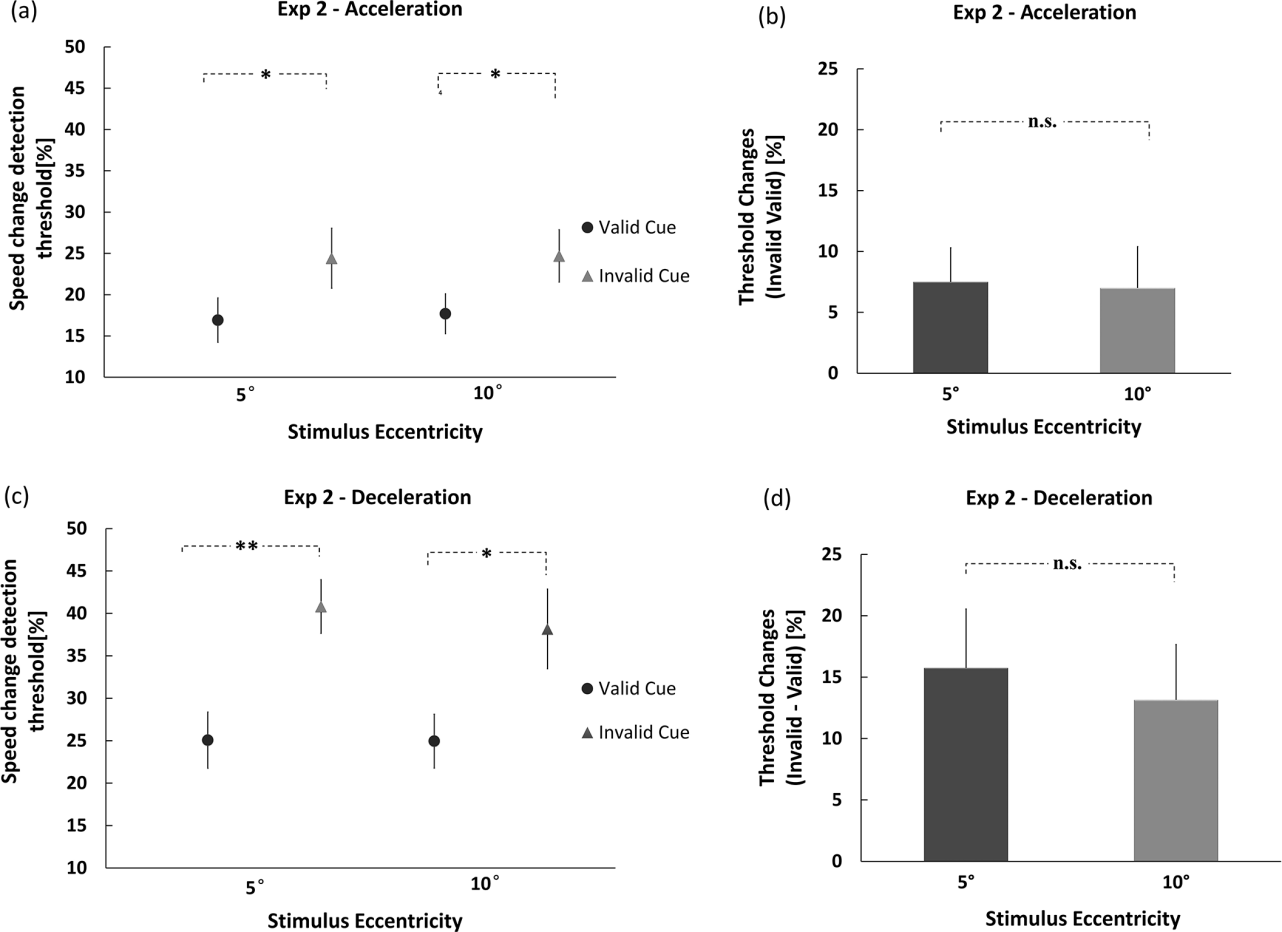
Effects of endogenous attention as indicated by speed-change thresholds in Exp 1. **(**a) Participants’ speed-change thresholds for acceleration in different cueing conditions (valid and invalid), at 5° and 10° eccentricity; (b) roughly equal amounts of endogenous cueing effects were observed for acceleration at 5° and 10°; (c) speed-change detection threshold for deceleration in the valid and invalid cueing conditions, at 5° and 10° eccentricity; (d) there was no difference between the cueing effects for deceleration at 5° and 10° eccentricities (* *p* < .05; ** *p* < .01; Error bars represent the standard error).

Participants’ detection thresholds (Fig 5A and 5C) were submitted to a three-way ANOVA, with stimulus eccentricity (5° or 10°) and cue-target validity (valid or invalid) as within-subjects variables, and sign of speed change (acceleration or deceleration) as between-subjects variable. The ANOVA produced two significant main effects only: one for cue validity (*F*(1,18) = 21.541, *p* < 0.001, $\eta_{p}^{2}$ = 0.545), again showing the cue-induced decrease of threshold at attended compared to unattended locations, and the other for sign of speed change (*F*(1,18) = 11.966, *p* < 0.01, $\eta_{p}^{2}$ = 0.399). The detection threshold for deceleration was significantly higher than that of acceleration (32.2% vs. 20.9%). Since the sign of speed change is a between-subjects variable, we further conducted two separate 2×2 ANOVAs, with eccentricity and cue validity as variables, one for acceleration and one for deceleration. The results demonstrated that the there was no two-way interaction between cue validity and stimulus eccentricity, neither for acceleration nor deceleration (*ps* > 0.05). The lack of interaction indicates that the attentional modulation was independent of stimulus eccentricity. Nevertheless, the cueing effects were reliably observed in both acceleration and deceleration conditions: when the speed change was positive, the magnitudes of attentional effects (unattended–attended) were 7.5% for 5°, and 7.0% for 10°eccentricity; when the speed change was negative, the cueing effects were 15.7% for 5° and 13.1% for 10°. No significant difference across eccentricity was obtained in either case (Fig 5B and 5D), which indicates endogenous cuing is not eccentricity-dependent.

## Discussion

The results of the present study revealed that participants performed better in detecting and making decisions about the speed change when the target appeared in cued rather than uncued locations with the exception of exogenous attention for deceleration in the perifoveal region. A novel result of our study was that exogenous attentional modulation had differential effects at the perifoveal compared to the near-peripheral regions of the visual field, whereas endogenous attention enhanced performance to the same extent at the two eccentricities.

Our results are consistent with previous psychophysical studies demonstrating that spatial attention enhances sensitivity for basic visual dimensions at the attended location, such as contrast sensitivity, spatial resolution, reaction time, two-pulse resolution, and many others [1,2,3,4,5,45,46]. One might classify these dimensions into being of an either more static or more dynamic nature [5]; the present study would then provide another example of attentional modulation of perceiving dynamic stimulus information. Indeed, studies in monkeys and humans have demonstrated a correlation between MT-neuron activity and performance during detection of changes in motion attributes, suggesting that neurons in this brain area play a key role in this type of task [47,48]. A recent study further provided evidence showing that the population transients in area MT were tuned to represent the sign and magnitude of the corresponding speed changes [11]. Moreover, attentional modulation of performance in motion tasks and the motion-generated neural activity has been widely observed in psychophysical and physiological studies [6,49,50]. Taken together, thus, one possible explanation for our observed attentional effect on motion-change detection could be that responses of MT neurons to a sudden speed change occur earlier and are stronger when the change is attended to as compared to unattended, thus increasing the ability of neurons to signal those changes. Indeed, recently it has been shown that attention can enhance the amplitude of the motion-change-evoked response, and shorten response latencies, in area MT of the macaque monkey [51]. Another study also reported that the response of MT neurons to the motion-direction change occurred at a shorter latency when the change was attended to as compared to unattended [52]. Thus, attention can modulate such change-evoked neuronal activities and thereby influence behavioral change-detection performance. One alternative explanation for the lower detection threshold at attended relative to unattended locations is that attention decreases response variability and thus improves the signal-to-noise ratio of the change-induced response [53,54]. Furthermore, it could also be possible that the decisional or response bias towards reporting a change in the cued stimulus leads to a reduction of the threshold at these locations. In order to dissociate different factors from sensitivity change leading to the observed attentional cueing effect, future studies will be mandatory to expand the current findings and provide better understanding on the mechanisms underlying the attentional modulation on speed-change perception.

A central question in the current study was whether the attentional modulation is homogenous across eccentricities. Here we used the magnitude of speed-change detection threshold as index and found a novel differential effect of eccentricity for exogeneous attention modulation. For detecting acceleration, a larger exogenous cueing effect indexed by a lower threshold for the cued relative to the uncued conditions was observed in the perifoveal region (5°) relative to the near-periphery (10°). While for perceiving deceleration, the pattern was reversed. To be precise, a large exogenous cueing effect was demonstrated in the near-periphery (10°), and no attentional modulation was observed in the perifoveal (5°) visual field. Interestingly, a recent psychophysical study also observed a similar differential effect for speed change itself, namely a higher foveal sensitivity (lower threshold) for detecting acceleration and a higher peripheral sensitivity for deceleration [12]. Thus, we speculate that there are differences in the way exogenous cueing effects are propagated across the visual field when the speed change is positive vs. negative, and that attention has a larger modulatory effect at spatial locations that are more sensitive to speed changes. This link to sensitivity may reflect the importance of the goal of attention, which is to improve estimates of stimulus properties.

But, how can the observed eccentricity effect of exogenous attention modulation be understood within a general frame taking an ecological perspective also into account? We would like to submit a speculative hypothesis which is based on some previous observations. It has been shown that the visual field shows inhomogeneity with respect to sensitivity, i.e. a central cone of foveal and perifoveal vision which is surrounded by a plateau of relatively constant sensitivity in the peripheral visual field [30]; this structural inhomogeneity is observed also under scotopic adaptation [55] when the central cone of foveal and perifoveal vision becomes less sensitive compared to the plateau region. This structural inhomogeneity is also reflected in attentional control as has been demonstrated with the eccentricity effect of IOR which has been proven to be a stable phenomenon [35,39,40].

In spite of this inhomogeneity there are at least three mechanisms that guarantee homogeneity of visual processing, i.e. constancy of brightness throughout the visual field for supra-threshold targets under photopic adaptation conditions [55,56], a common time window of approximately three seconds when the time course of perifoveal and peripheral inhibitory effects of spatial attention is investigated [57], and on a more theoretical level the integration of sensory processing and motor control as it is hypothesized with the reafference principle [58]. As has been stated previously, within this theoretical account, different attentional fields represent service operations for an optimal processing of visuo-motor actions. Homogeneity of visual space is a necessary condition for an unbiased selection of potential visual targets; inhomogeneity of visual space reflects different mechanisms that are necessary for efficient action.

In the experiments on speed change perception reported here stimuli were presented at 5° or 10° eccentricities of the visual field. Based on the observations mentioned above it can be assumed that stimuli in these two stimulus positions are predominantly processed in two processing streams, i.e. one with the emphasis in the retino-geniculo-striate projection system (5° eccentricity), and one with the retino-colliculo-extrastriate projection system (10° eccentricity), although the latter is located in the border region of the two projection systems. However, as the experiments were done with a viewing distance of 57 cm (see methods), the visual axes of the two eyes had to converge by some degrees and also accommodation of the lenses were necessary which contribute in the near-distance space to size constancy [59]; these factors have been shown to modulate perifoveal sensitivity, i.e. modulating the transition zone between the central cone of foveal and perifoveal vision and the plateau region. Thus, we believe that in our experiments we are dealing with operational principles emphasizing the two anatomically different processing streams.

Embedded in the above general frame, how to understand the differential exogenous attention modulation in perifoveal and near-peripheral regions for detecting acceleration and deceleration respectively? Here we would like to further submit an ecological account with respect to attentional control in the visual space. As the perifoveal region and the periphery are typically involved in different cognitive functions, namely, object perception vs. spatial navigation, acceleration and deceleration of moving objects might trigger very different attentional processing modes. In the condition of acceleration, the speeded moving target might mimic an escaping prey being chased after. Therefore, a focused allocation of spatial attention is typically triggered, no matter where the target is. As attention can facilitate perception at the attended location, it is not surprising that we observed exogenous cueing effects at both perifoveal and peripheral locations. But why the cueing effect is larger at perifoveal relative to peripheral locations? This might be related to the different “attentional fields” in the visual field.

In research on the structural involvement of midbrain mechanisms in attentional control the size of attentional fields at different locations in the visual field could be inferred [60]. In those experiments the paradigm of central fatigue or habituation was employed when measuring light-difference thresholds at different locations in the visual field. It has been shown that continuous measurement of thresholds at one location results in habituation, i.e. the sensitivity decreases substantially and it takes ca. 20 minutes or so for spontaneous recovery. As habituation can be observed after monocular stimulation also in the non-stimulated eye, the habituation must be of central origin reflecting fatigue at the cortical level, and one is not dealing with retinal adaptation. The surprising effect reported by Singer and colleagues [60] is that habituation can be cancelled instantaneously if a mirror-symmetric position is stimulated. As spontaneous recovery takes a much longer time (20 minutes or so), an inter-hemispheric interaction has to be assumed. As the instantaneous resetting of threshold occurs also when an area is stimulated in a blind region (scotoma) of the visual field in patients who have suffered an injury in striate cortex, it can be concluded that the interaction between the two hemispheres takes place at the midbrain level [61]. In these experiments on resetting of thresholds it was possible to determine the spatial extent of the resetting area. It turned out that the retinal areas which contributed to such resetting were not limited to the exact retinal position possibly reflecting the diameter of receptive fields, but they were much larger and could be defined as "attentional fields". At 5° eccentricity the diameter of the attentional field is approximately 6° visual angle; at 40° eccentricity it is approximately 20° visual angle. Thus, a gradual increase of the size of attentional field from the perifoveal region to the periphery can be assumed. Although acceleration condition in the present study may trigger similar focused allocation of spatial attention to the cued location, the size of attentional field might be smaller for the 5° relative to the 10° eccentricity, thus leading to stronger attention power at the less eccentric location. As a consequence, a larger attention effect was observed for 5° relative to 10° eccentricity.

In the condition of deceleration, which perhaps signals potential danger such as a predator approaching us in a natural environment, we would like to propose that a divided attention mode is more likely to be triggered. However, divided attention to both visual fields is only possibly within a relatively small visual space such as within the perifoveal visual field and not beyond. This may explain why we did not observe any attention effect for the 5° eccentricity in the deceleration condition, since subjects’ attention were equally distributed to both the cued and the uncued locations without any attentional bias. Since for 10° eccentricity the divided attention mode is presumably not possible and the subjects still have to activate the focused allocation of attention, the observation of an attention effect, i.e., a lower speed change threshold at the cued relative to the uncued location, is consistent with the activated attentional mode being triggered in such condition, as we speculate from an ecological perspective.

Note, however, that the sign of speed change was designed as a between-subjects factor, such that eccentricity effects observed in acceleration and deceleration conditions for the same stimulus eccentricity are not directly comparable. Future work using a full within-subject design will be particularly informative in disentangling this differential eccentricity effect of involuntary attention with respect to positive and negative speed changes.

Results from Exp 2 showed that endogenous attention also affects speed-change perception, but that, unlike in exogenous attention, the effects do not vary with eccentricity in the range measured. Directing endogenous attention via a foveal, informative cue to the target location improved speed change detection by the same amount at the two eccentric locations. This result suggests that top-down modulation is organized similarly in these regions. A recent study that manipulated either exogenous or endogenous attention in foveal and perifoveal locations (1° vs. 7°) has shown that exogenous attention had a larger cueing effect on reaction times for the perifovea than for the fovea, whereas endogenous attention improved performance at both eccentricities equally [62]. In a study by Yeshurun and colleagues [63] exogenous attention improved performance in a texture segmentation task at far eccentricities and impaired it at near eccentricities, whereas endogenous attention improved performance at all measured eccentricities. Together with the present findings, the most immediate inference appears to be that endogenous attention, being of top-down nature, can be consciously controlled and evenly distributed over the visual field, whereas exogenous attention relies on more hardwired, low-level mechanisms that operate differently in different regions of the central visual field. A next step will be to find out whether the observed dissociation between acceleration and deceleration for exogenous, but not for endogenous, attention holds up for other, more peripheral locations in the visual field.

In summary, while there is ample evidence for eccentricity-dependent modulation of spatial attention, the present study represents the first investigation of the attentional effects on speed-change perception in different locations in the visual field. The results suggest that exogenous attentional modulation has differential effects between the perifoveal and near-peripheral regions of the visual field, whereas endogenous modulation is homogeneous within the central 10°-radius field. Since these two types of attention have distinct effects on performance, they are most likely mediated by different mechanisms.

## Supporting information

S1 Table Percent correct for each subject under acceleration condition in Exp 1. (XLSX)

S2 Table Percent correct for each subject under deceleration condition in Exp 1. (XLSX)

S3 Table Thresholds for each subject in Exp 1. (XLSX)

S4 Table Percent correct for each subject under acceleration condition in Exp 2. (XLSX)

S5 Table Percent correct for each subject under deceleration condition in Exp 2. (XLSX)

S6 Table Thresholds for each subject in Exp 2. (XLSX)
